# Incidence and risk factors for acute kidney injury following autologous stem cell transplantation for multiple myeloma

**DOI:** 10.1002/cam4.2187

**Published:** 2019-04-23

**Authors:** Andreea G. Andronesi, Alina D. Tanase, Bogdan M. Sorohan, Oana G. Craciun, Laura Stefan, Zsofia Varady, Lavinia Lipan, Bogdan Obrisca, Alexandra Truica, Gener Ismail

**Affiliations:** ^1^ Nephrology Department Fundeni Clinical Institute Bucharest Romania; ^2^ Carol Davila University of Medicine and Pharmacy Bucharest Romania; ^3^ Bone Marrow Transplant Department Fundeni Clinical Institute Bucharest Romania

**Keywords:** acute kidney injury, multiple myeloma, stem cell transplant

## Abstract

Acute kidney injury (AKI) is a common complication after allogeneic stem cell transplantation; however, its incidence and outcome in patients transplanted for multiple myeloma (MM) is unknown. We evaluated the incidence, severity, and risk factors for AKI within the first 30 days after autologous stem cell transplantation (ASCT) for MM. We prospectively followed 185 consecutive patients with MM, without chronic renal replacement therapy, who underwent ASCT; 12.5% of patients had MM‐associated amyloidosis. AKI occurred in 19 (10.3%) patients, 8 ± 3 days after ASCT, with 18 patients (9.7%) stage 1 and one patient (0.6%) stage 2 AKI. The development of AKI was not associated with reduced overall survival and recovery of kidney function was evident in 68.4% of patients at 3 months. In Cox regression analysis, preexisting–chronic kidney disease (HR 7.01, CI 95% 2.04‐24.09; *P* = 0.002), serum beta2 microglobulin (HR 3.05, CI 95% 1.10‐8.44; *P* = 0.03), and mucositis grade 3/4 (HR 1.29, CI 95% 1.08‐1.53; *P* = 0.003) were independent risk factors for AKI. Our results suggest that AKI occurs with low incidence and reduced severity after ASCT for MM. Prophylactic measures in patients with preexisting–kidney failure may further reduce this risk.

## 1 INTRODUCTION

Multiple myeloma (MM) is the most frequent malignant monoclonal gammopathy and the second most common hematological malignancy after non‐Hodgkin lymphoma.^1^ The annual incidence rate for US was estimated to be 6.5 per 100 000 for 2010‐2014 with a death rate of 3.3 per 100 000 for 2011‐2015.^2^ A similar incidence was reported for Europe,^3^ with a stable incidence over the past six decades.^4^ A better understanding of disease biology and therefore of risk stratification according to genetic changes, together with introduction of novel therapies such as proteasome inhibitors, immunomodulators, and hematopoietic stem cell transplantation were major steps in MM management during the past 10 years. Compared to standard chemotherapy alone, myeloablative conditioning consisting of high‐dose intravenous melphalan followed by autologous stem cell transplantation (ASCT) in eligible patients offers better long‐term cancer‐free and overall survival.^5^ Kidney involvement is a prominent feature of MM and is mainly due to high‐tumor burden with development of myeloma cast nephropathy, but also because of hypercalcemia and tumor lysis syndrome. Kidney failure is a negative prognostic factor for overall survival in MM and may impair eligibility for ASCT especially for advanced stages of chronic kidney disease (CKD), although similar outcomes were observed for milder and moderate CKD stages when compared to normal kidney function.^6^ Stem cell transplantation (SCT) is a major advance in treatment of various hematologic malignancies disorders (including MM) and bone marrow insufficiencies, but its use is limited by important complications, including acute kidney injury (AKI). Incidence of AKI is estimated to be between 10% and up to 75% during the first 100 days after transplantation and is influenced by the definition used in various studies, type of transplant (autologous or allogeneic), and type of conditioning (myeloablative or non‐myeloablative).^7^ Up to 5% of these patients need acute dialysis^8^ and the severity of AKI is associated with poorer short‐ and long‐term outcomes.^9^ Nevertheless, even milder, more “benign” increase in serum creatinine (up to 0.5 mg/dL) is associated with longer hospitalization and reduced survival.^10^ A higher incidence of AKI is observed in allogeneic SCT compared to ASCT because of medication used (calcineurin inhibitors, amphotericin, acyclovir, cyclophosphamide, methotrexate), occurrence of graft versus host disease, or hepatic sinusoidal obstruction syndrome. The purpose of the present study was to investigate the incidence and risk factors for AKI development during the first 30 days following ASCT for MM at our institution. To the best of our knowledge, this is the first study in the medical literature addressing this kidney complication after ASCT for this specific hematologic malignancy.

## 2 METHODS

### 2.1 Study design

We performed a prospective non interventional study in which we enrolled 185 consecutive patients with MM, not on chronic renal replacement therapy, who underwent ASCT between January 2016 and December 2017. All patients were followed for 90 days starting from the day of ASCT (baseline). The aims of our study were incidence of AKI within the first 30 days after ASCT and identification of risk factors for AKI occurrence.

The study was conducted in accordance with the Declaration of Helsinki human rights and was approved by the Institutional Review Board of Fundeni Clinical Institute. All the patients signed the informed consent.

### 2.2 Autologous stem cells transplantation

Eligibility criteria for ASCT included age between 18 and 75 years, prior psychiatric evaluation, no severe major organ failure (lung, heart, liver), no active infections, no uncontrolled diabetes mellitus, and performance status ≤2 according to Eastern Oncology Cooperative Group (ECOG).^11^ There was no limitation by kidney function. Prior to ASCT, patients were treated with four to six series of chemotherapy according to local protocols (CyBorD, cyclophosphamide, bortezomib, dexamethasone or PAD, bortezomib, doxorubicin, dexamethasone). The source of hematopoietic progenitor cells was peripheral stem cells. Stem cell grafts were harvested after mobilization with granulocyte‐colony stimulating factor G‐CSF (filgrastim) 10 to 16 μg/kg, or after cyclophosphamide 3 g/m^2^ followed by G‐CSF. Myeloablative standard conditioning with melphalan intravenously in single dose was used. Melphalan dose (140 or 200 mg/m^2^) was established per protocol according to age, kidney function, and performance status. Stem cells were infused 48 hours after conditioning with daily administration of G‐CSF (5 mg/kg) starting day +5. Prophylactic antibiotics and antivirals (ciprofloxacin and acyclovir) were given to all patients. Broad‐spectrum antibiotics were used for febrile neutropenia as per institutional protocol.

### 2.3 Collected data and follow‐up

Clinical and laboratory data were collected at baseline, except serum beta2 microglobulin (β2MG) and serum free κ and λ light chains which were measured prior to the myeloablative conditioning.

Gender, age, and type of MM were recorded for all patients. Data regarding previous ASCT and comorbidities (diabetes mellitus, hypertension, congestive heart failure, CKD, AKI) were also recorded. The estimated glomerular filtration rate (eGFR) was estimated using the CKD‐EPI formula.^12^

During the study period, the patients were examined and blood for laboratory tests (including serum creatinine for eGFR assessment) was analyzed at least daily until engraftment and at day + 30 after ASCT. In patients with AKI, eGFR was evaluated also after 90 days following AKI episode. Discharge criteria were WBC >1500/mm^3^ and PLT > 20 000/mm^3^.

### 2.4 Definitions

AKI within the first 30 days after transplantation was defined and graded according to the KDIGO definitions and classification systems^13^; the highest stage of AKI occurring within the first 30 days after ASCT was used in analysis.

Renal recovery was defined as a reduction in serum creatinine to less or within 0.3 mg/dL as compared to baseline occurring at 3 months following the AKI diagnosis.

The diagnosis of MM and the assessment of response to therapy were made according the International Multiple Myeloma Working Group (IMWG) Criteria^14^, ^15^ and MM staging, according to the Durie Salmon staging system.^16^ The associated‐AL amyloidosis was diagnosed by demonstration of Congo red staining (kidney, subcutaneous fat or rectal mucosa biopsy). Hypertension (HBP) before transplantation was defined as a documented history of hypertension, the use of antihypertensive drugs, or a blood pressure of over 140/90mm Hg on the day of admission for ASCT. CKD was defined using the Kidney Disease: Improving Global Outcomes (KDIGO) organization's 2012 guidelines.^17^ Sepsis diagnosis was based on a temperature ≥38°C or <36°C, a white blood cell count >10 000/mm^3^ or <4000/mm^3^, and a positive blood culture for bacteria.^18^ Oral mucositis was graded according to World Health Organization's (WHO's) Oral Toxicity Scale.^19^

### 2.5 Statistical analysis

Data are reported as percentages for categorical data, mean ± standard deviation for continuous parametric data and median with interquartile range for continuous nonparametric data. The cohort was divided in two groups namely: AKI and non‐AKI. The differences between groups were assessed using Student *t* test for normally distributed continuous variables, Mann‐Whitney *U* for non‐normally ones and chi‐squared and Student *t* tests for categorical variables. Kaplan‐Meier curves were used to evaluate the cumulative probability of AKI incidence and the risk associated with several factors was analyzed by Log‐rank test. To identify the predictors associated with the risk of AKI, Cox proportional hazard analysis was used. In univariate analysis the variables with *P* values less than 0.10 at groups' comparison were introduced. In multivariate analysis model, we introduced all the variables from univariate analysis and we applied the stepwise backward elimination process. Covariates included in the Cox model can be classified as clinical‐related (baseline eGFR, history of AKI, preexisting–CKD, hypertension), hematological‐disease related (micromolecular MM, MM and amyloidosis, lambda light chain, serum β2M, MM stage IIIB), and treatment‐related (ACE‐ inhibitors/ARB, mucositis grade 3/4).

Sensitivity, specificity, positive predictive value (PPV), negative predictive value (NPV), positive likelihood ratio (positive LR), negative likelihood ratio (negative LR), and accuracy were calculated to test the prediction ability for AKI of the independent determinants identified in the Cox model.

A *P* < 0.05 was considered statistically significant.

Statistical analysis was made with IBM SPSS Statistics, Version 21 (SPSS Inc, Chicago, IL) and STATA Version 14.

## 3 RESULTS

### 3.1 Characteristics of the investigated cohort

ASCT was performed between January 2016 and December 2017 in 185 patients, for the first and second time in 155 (83.8%) and 30 (16.2%), respectively. Mean age at day 0 was 55.6 ± 8.5 years and 50.3% were females (Table 1).

**Table 1:** Investigated patients' characteristics

	Overall	No AKI	AKI	*P*
Patients' number	185	166	19	
Demographic data				
Age (mean, years)	55.6 ± 8.5	55.5 ± 8.6	56.1 ± 7.5	0.78
Male gender (%)	92 (49.7%)	82 (49.4%)	11 (57.9%)	0.48
Comorbidities				
Hypertension (%)	81 (43.8%)	69 (41.6%)	12 (63.2%)	0.07
CHF (%)	15 (8.1%)	13 (7.8%)	2 (10.5%)	0.69
Type 2 DM (%)	28 (15.1%)	23 (13.9%)	5 (26.3%)	0.18
History of AKI (%)	11 (5.9%)	8 (4.8%)	3 (15.8%)	0.09
Preexisting–CKD (%)	27 (14.6%)	13 (7.8%)	14 (73.7%)	<0.001
ACE inhibitors/ARB (%)	43 (23.2%)	35 (21.1%)	8 (42.1%)	0.05
Kidney function				
eGFR (mean, ml/min)	86.1 ± 24.1	90.1 ± 20.8	51.8 ± 25	<0.001
Hematologic disease				
Type (%)				0.01
Non‐secretory MM	2 (1.1%)	2 (1.2%)	0 (0%)	
Micromolecular MM	47 (25.5%)	40 (24.1%)	7 (36.8%)	
IgM MM	2 (1.1%)	2 (1.2%)	0 (0%)	
IgG MM	96 (51.3%)	90 (54.2%)	5 (26.3%)	
IgD MM	3 (1.6%)	2 (1.2%)	1 (5.3%)	
IgA MM	34 (18.4%)	30 (18.1%)	4 (21.1%)	
Amyloidosis	2 (1.1%)	0 (0%)	2 (1.2%)	
MM and amyloidosis (%)	23 (12.5%)	17 (10.2%)	6 (31.6%)	<0.001
MM stage (%)^a^				<0.001
IA	2 (1.1%)	2 (1.3%)	0 (0%)	
IIA	12 (6.5%)	10 (6.3%)	2 (12.5%)	
IIB	2 (1.1%)	1 (0.6%)	1 (6.2%)	
IIIA	122(65.9%)	121 (76.1%)	1 (6.2%)	
IIIB	37 (20%)	25 (15.7%)	12 (75%)	
Bone marrow plasma cells (median, %)	6 (4‐14)	6 (4‐13)	11 (3.7‐46.2)	0.18
Serum free κ light chain (median, mg/L)	4.8 (2.1‐18)	4.5 (1.8‐18)	12.9 (4.6‐18.5)	0.15
Serum free λ light chain (median, mg/L)	3.7 (1.8‐ 14.7)	3.4 (1.6‐ 14.1)	14.1 (3.8‐250)	0.008
Serum β2M (median, mg/L)	2.6 (2.2‐3.6)	2.9 (2.1‐3.5)	5.9 (3.2‐9)	<0.001
Previous ASCT (%)	30 (16.2%)	27 (16.3%)	3 (15.8%)	0.95
Complications				
Mucositis grade 3/4 (%)	57 (30.8%)	45 (27.1%)	12 (63.2%)	0.01
Fever (%)	111 (60%)	96 (57.8%)	15 (78.9%)	0.12
CD enteritis (%)	7 (3.8%)	7 (4.2%)	0 (0%)	0.21
Sepsis (%)	31 (16.8%)	26 (15.7%)	5 (26.3%)	0.26
Death (%)	2 (1.1%)	1 (0.6%)	1 (5.3%)	0.01

ACEI, angiotensin converting enzyme inhibitors; AKI, acute kidney injury; ARB, angiotensin receptor blocker; ASCT, Autologous stem cells transplant; β2M‐ beta 2 microglobulin; CD, clostridium difficile; CKD, chronic kidney disease; CHF, cardiac heart failure; DM, diabetes mellitus; eGFR, estimated glomerular filtration rate; MM, multiple myeloma

a missing data in 10 patients

Preexisting–CKD was noted in 14.6% (mean eGFR at baseline 86.1 ± 24.1 ml/min), 5.9% had a history of AKI and 15.1% type 2 diabetes mellitus. HBP was present in 43.8% and 23.2% were treated with angiotensin converting enzyme inhibitors (ACEI) or angiotensin receptor blockers (ARB).

Median hematologic disease duration before ASCT was 10 months (8‐18), IgG MM was the most frequent (51.3%) and the most common MM stage was IIIA (65.9%).

AKI occurred in 19 (10.3%) patients, mean time of appearance was 8 ± 3 days after ASCT, 18 patients (94.7%) out of 19 had AKI stage 1 and one patient (5.3%) had AKI stage 2.

Regarding complications after ASCT, fever was the most frequent one (60%), followed by grade 3/4 mucositis (30.8%). Two patients died during the study, at day + 35, and at day + 51 respectively after ASCT.

### 3.2 Comparison between patients with and without AKI

AKI patients had more frequently preexisting–CKD (73.7% vs 7.8%) and a significantly lower eGFR (51.8 ± 25 vs 90.1 ± 20.8 mL/min) at baseline. Also, they had a trend to a more frequent history of previous episodes of AKI (15.8% vs 4.8%, *P* = 0.09), hypertension (63.2% vs 41.6%, *P* = 0.07) and ACEI/ARB use (42.1% vs 21.1%, *P* = 0.05) (Table 1).

Patients in the AKI group had significantly more frequent micromolecular MM (36.8% vs 24.1%) in advanced stages (IIIB) (75% vs 15.7%) and associated amyloidosis in a higher proportion (31.6% vs 10.2%). The serum free λ light chain and β2M levels were higher in this group (14.1 vs 3.4 mg/L and 5.9 vs 2.9 mg/L, respectively) (Table 1). No differences in the risk for AKI occurrence was found between the two chemo regimens used prior to transplantation.

### 3.3 Predictors of AKI

**Figure 1:**
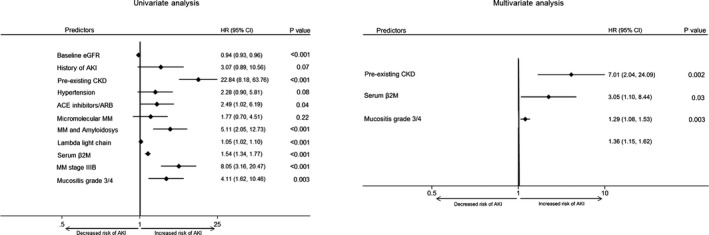
Multivariate Cox model *(right)* with stepwise backward elimination process: variables introduced in the first step (baseline eGFR, history of AKI, preexisting–CKD, hypertension, ACE‐ inhibitors/ARB, micromolecular MM, MM and amyloidosis, lambda light chain, serum β2M, MM stage IIIB, mucositis grade 3/4), variables remained in the final step (preexisting–CKD, serum β2M, mucositis grade 3/4); *P* < 0.05, statistically significant. HR, hazard ratio; eGFR, estimated glomerular filtration ratio; AKI, acute kidney injury; CKD, chronic kidney disease; ACE, angiotensin converting enzyme; ARB, angiotensin receptor blockers; MM, multiple myeloma; β2M, β2 microglobulin

All the variables with a *P* values less than 0.10 at groups' comparison were analyzed in a Cox regression model. A more severe hematologic disease (MM stage IIIB, MM associated with amyloidosis, higher serum free λ light chain and β2M levels), kidney condition (baseline eGFR, preexisting–CKD, and ACEI or ARB therapy), and more frequent ASCT complications (mucositis grade 3 or 4) were significantly associated with AKI in univariate analysis. In multivariate Cox regression analysis, preexisting–CKD (HR 7.01, CI 95%: 2.04‐24.09; *P* = 0.002), serum β2M (HR 3.05, CI 95%: 1.10‐8.44; *P* = 0.03) and mucositis grade 3/4 (HR 1.29, CI 95%: 1.08‐1.53; *P* = 0.003) were retained as independent risk factors for AKI (Figure 1).

**Figure 2:**
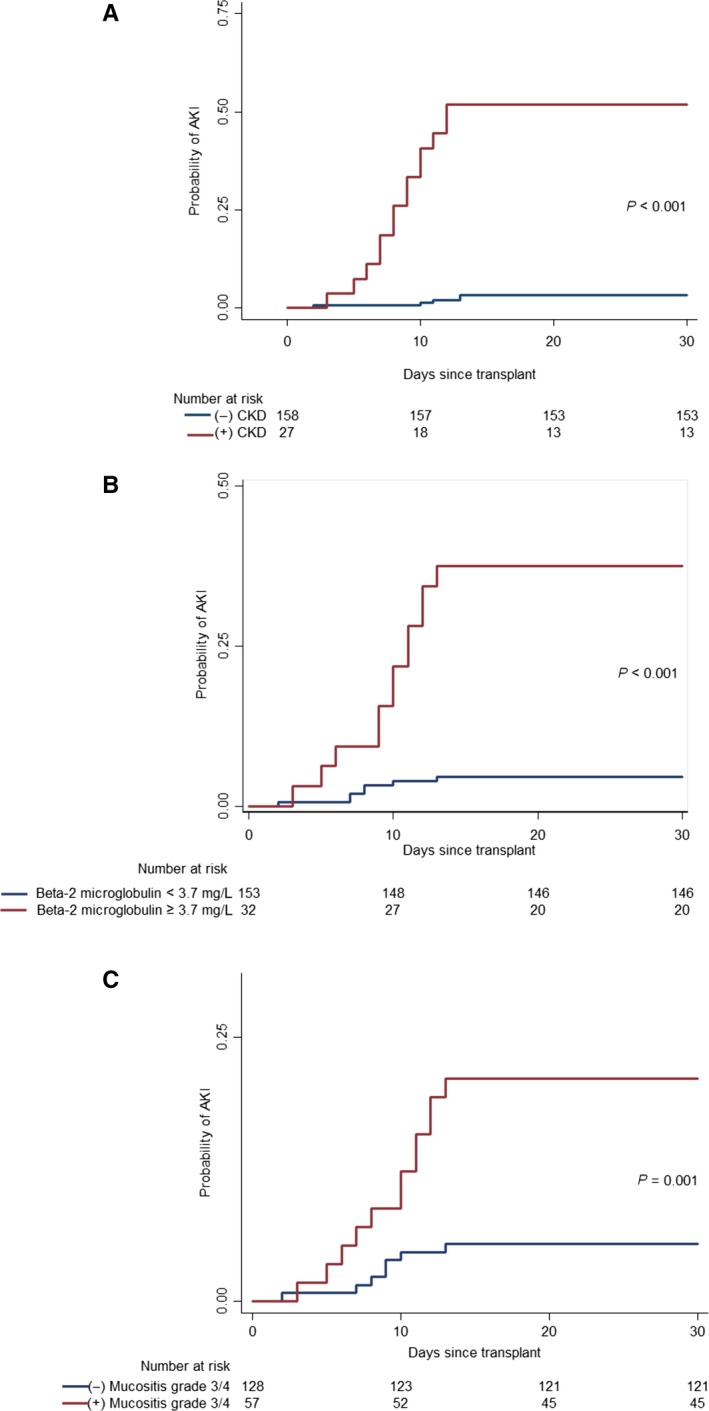
Time‐to‐event curves for AKI differed significantly between preexisting and non‐preexisting–CKD (Panel A), serum β2M ≥ 3.7 mg/L and serum β2M < 3.7 mg/L (Panel B), and between mucositis grade 3/4 and non‐mucositis grade 3/4 patients (Panel C) (*P* < 0.001, *P* < 0.001 and *P* = 0.001, respectively, by the log‐rank test). CKD, chronic kidney disease; β2M, β2 microglobulin; AKI, acute kidney injury

In Kaplan‐Meier analysis, the cumulative probability of AKI in the first 30 days after ASCT was higher in patients with preexisting–CKD (52% vs 3%; *P* < 0.001), serum β2MG ≥ 3.7 mg/L (38% vs 5%; *P* < 0.001), and developing severe (grade 3/4) mucositis (21% vs 7.3%; *P* < 0.001) **(**Figure 2).

The predictive ability for AKI in the first 30 days of these three independent risk factors was further evaluated. Preexisting–CKD had the best accuracy (90.3%, 95% CI 85%‐94.1%), followed by serum β2M level ≥ 3.7 mg/L (85.4%, 95% CI: 79.5%‐90.1%) and mucositis grade 3/4 (71.9%, 95% CI: 64.8%‐78.2%) (Table 2).

**Table 2:** The predictive utility for AKI after ASCT of preexisting–CKD, serum β2MG and severe mucositis

	Sensitivity	Specificity	PPV	NPV	Accuracy
Preexisting–CKD	73.6% (48.8%‐90.8%)	92.1% (86.7%‐95.7%)	51.8% (37.4%‐65.9%)	96.8% (93.5%‐98.5%)	90.3% (85%‐94.1%)
Serum β2M ≥ 3.7 mg/L	63.1% (38.3%‐83.7%)	87.9% (82%‐92.5%)	37.5% (26%‐50.6%)	95.4% (92%‐97.4%)	85.4% (79.5%‐90.1%)
Mucositis grade 3/4	63.1% (38.3%‐83.7%)	72.9% (65.4%‐79.5%)	21% (14.8%‐28.9%)	94.5% (90.5%‐96.9%)	71.9% (64.8%‐78.2%)

PPV, positive predictive value; NPV, negative predictive value; CKD, chronic kidney disease; β2M, beta2 microglobulin

## 4 DISCUSSION

It is estimated that more than 50 000 hematopoietic stem cell transplants are performed annually worldwide.^7^ Although a lifesaving procedure, it is associated with important side effects, and AKI is one of the most important in terms of raising medical costs, but especially in altering patients' outcome. Incidence of AKI in allogeneic SCT is higher compared to ASCT, mostly because of calcineurin inhibitors, occurrence of graft versus host disease and hepatic sinusoidal obstruction syndrome, which are important risk factors for AKI in this setting. The common use of peripheral stem cells instead of bone marrow cells, which reduces the time span till engraftment and thus the risk for sepsis is another explanation for lower incidence for AKI in ASCT. The incidence of AKI following myeloablative conditioning and ASCT ranges from 12%^20^ to as high as 52%,^21^ with 5%‐16% of patients requiring acute dialysis.^20^, ^22^

We found that 10% of our patients with MM treated with ASCT developed AKI between the time of infusion of stem cells (day 0) and 30 days after the transplant. None of our patients needed initiation of dialysis and recovery of kidney function was evident in 68.4% of patients at 3 months after the onset of AKI. Even in our study, mortality was significantly higher in subgroup of patients with AKI (*P* = 0.01), we cannot translate this finding into clinical practice due to the very small number of deaths which occurred during the study period. In a retrospective analysis of 173 patients with AL amyloidosis treated with ASCT, AKI occurred in 21% of patients, 5% needed acute dialysis and AKI was associated with reduced survival 90 days after transplant.^22^ The differences in incidence and severity between our study and the results of Fadia et al may be due to the fact that AL amyloidosis is a systemic disease, with frequent involvement of the kidney and heart, preexisting conditions which increase the likelihood of kidney injury after ASCT. It is estimated that around 10% of patients with MM have coexisting AL amyloidosis.^23^, ^24^ In our cohort of patients, AL amyloidosis secondary to MM was diagnosed in 12.5% of patients and coexistence of these two conditions was associated with a fivefold higher risk of AKI following ASCT.

MM differs from other hematologic malignancies treated by bone marrow transplantation in that a significant proportion of patients already have important irreversible kidney lesions prior to transplantation due to intrinsic nephrotoxicity of monoclonal proteins. A total of 14.6% of our patients had preexisting–CKD and this condition was the most important independent predictive risk factor for AKI during the first 30 days after ASCT, with a sevenfold increase in the likelihood of developing AKI. Also, the severity of preexisting–CKD was a risk factor for AKI (event incidence was 8.6% in those without CKD as compared with 45% in mild and moderate CKD and 71.4% in advanced CKD). Underlying CKD is now a well‐recognized risk factor for AKI (acute‐on‐chronic kidney insufficiency). CKD was identified as an independent risk factor for developing AKI in the setting of different conditions such as sepsis, radiocontrast agents, cardiovascular surgery, liver failure, or exposure to nephrotoxins (chemotherapeutic agents, antibiotics).^25^ The incidence of AKI complicating CKD varies depending on the definitions used for AKI and CKD and the type of population analyzed, ranging from 10% in the general population‐based studies up to 35% in the hospital‐based studies.^26^ Increased risk for AKI becomes evident even in patients considered to have “mild” CKD, corresponding to a GFR between 30‐59 mL/min/1.73 m^2^, and this risk becomes progressively more important from stage 3 to stage 5 CKD, independent of other comorbidities.^27^ Hsu et al compared 1746 hospitalized patients from Kaiser Permanente who developed severe AKI requiring dialysis with 600 820 hospitalized patients who did not develop AKI; patients with stage 3a CKD had a twofold higher risk for AKI compared to the patients with GFR above 60 mL/min/1.73 m^2^ and this risk became even higher in more advanced CKD (up to a 40‐fold increased risk for those in stage 5).^27^ We found similar results, since baseline GFR was a risk factor for AKI and, when compared with patients with normal GFR, even patients with mildly impair of kidney function (GFR between 60‐89 ml/min/1.73 m^2^) had an adjusted 11.2‐fold increased risk of developing AKI after ASCT. Creatinine clearance was also found by Fadia et al as independent predictor factor for AKI after ASCT for AL amyloidosis.^22^ Multiple, yet not fully understood, mechanisms explain the higher risk for AKI in CKD patients; reduced kidney reserve, altered vasodilatation, a higher diabetes and hypertension incidence in CKD conditions also proved to be risk factors for different types of AKI.^28^

The fact that we observed that 73% of all AKI events occurred among patients with some degree of already impaired kidney function means that careful kidney function surveillance and prophylactic measures (such as maintaining euvolemia and avoiding nephrotoxins) should be undertaken during the first weeks after ASCT. Failing kidneys have a reduced capacity to respond to hypovolemia because of inability to concentrate urine, and this defect puts patients with CKD at a higher risk of developing prerenal azotemia because of dehydration.^29^ We found an adjusted 30% increased risk of developing AKI for patients with mucositis grade 3 or 4, an important cause of extracellular volume depletion because of vomiting and diarrhea. Moreover, treatment with ACEI or ARB was associated with more than twofold higher risk of developing AKI, although this association did not remain statistically significant in multivariate analysis. Autoregulation is important for maintaining glomerular filtration despite significant drop in blood pressure due to dehydration and this mechanism is altered in CKD and may be further impaired in patients treated with agents which interfere with renin‐angiotensin‐aldosterone system, such as ACEI or ARB.^30^

In addition to clinical data, we found serum beta2 microglobulin as an independent predictor of AKI after ASCT and that for every increase in baseline β2M with 1 unit above 3.7 mg/L the risk for AKI increases by 3‐fold. To date, none of the previous studies regarding AKI after SCT focused on β2M. Serum β2M is a marker of tumor load in lymphoproliferative disorders (including MM) and of kidney insufficiency, both important predictors of outcome in MM.^31^ Together with albumin, serum β2M is a key factor in current International Staging System,^32^ but it also predicts progression from smoldering‐MM to symptomatic disease, response to chemotherapy, relapse, and even prognosis after transplantation.^33^, ^34^, ^35^ This is the first study in which β2M was also found to be an independent prognostic factor of AKI following ASCT for MM. Along with the tumor burden, β2M is also a marker of kidney function.^36^ A better hematological response before ASCT means a lower serum level of nephrotoxic monoclonal proteins and thus a reduced kidney susceptibility to other potentially harmful insults.

Our study has several limitations. It is a single‐center study, so our findings may not be generalized to all patients. The relatively small number of AKI events that we encountered may reduce the statistical power to identify other important risk factors, so our results need to be validated in larger and multicenter cohorts of patients. However, our study has some important strengths, the most important one being the large size of the cohort and the prospective nature of the design.

In summary, although it does not produce a definitive cure, ASCT is now considered the standard of care for eligible patients with MM, significantly prolonging both event‐free and overall survival when compared to standard chemotherapy alone.^37^ Compared to what was found in studies dedicated to SCT for other types of hematological conditions, including AL amyloidosis, we found a much lower incidence and reduced severity of AKI in patients transplanted for MM. These are positive results since it seems that AKI occurrence should not be such a big concern in this particular type of hematological malignancy. Our results regarding potential risk factors may also improve individual risk stratification and proper choose of prophylactic measures, with greater emphasis on avoiding dehydration and further exposure to nephrotoxins especially in patients with previous kidney insufficiency.

## AUTHOR CONTRIBUTIONS

AGA, BMS, BO, and GI conceptualized the research and formed the hypothesis of this paper. AGA, AT, ADT, OGC, LS, ZV, and LL collected the data. BMS and GI analyzed the data. AGA, BMS, GI, and ADT wrote the manuscript. All the authors have critically evaluated the manuscript and approved the final version.
